# Additional Drug Resistance of Multidrug-Resistant Tuberculosis in Patients in 9 Countries

**DOI:** 10.3201/eid2106.141329

**Published:** 2015-06

**Authors:** Ekaterina V. Kurbatova, Tracy Dalton, Julia Ershova, Thelma Tupasi, Janice Campos Caoili, Martie Van Der Walt, Charlotte Kvasnovsky, Martin Yagui, Jaime Bayona, Carmen Contreras, Vaira Leimane, Laura E. Via, HeeJin Kim, Somsak Akksilp, Boris Y. Kazennyy, Grigory V. Volchenkov, Ruwen Jou, Kai Kliiman, Olga V. Demikhova, J. Peter Cegielski

**Affiliations:** Centers for Disease Control and Prevention, Atlanta, Georgia, USA (E.V. Kurbatova, T. Dalton, J. Ershova, J. Campos Caoili, C. Kvasnovsky, J.P. Cegielski);; Tropical Disease Foundation, Manila, the Philippines (T. Tupasi, J. Campos Caoili);; Medical Research Council, Pretoria, South Africa (M. Van Der Walt, C. Kvasnovsky);; National Institute of Health, Lima, Peru (M. Yagui); Socios en Salud Sucursal Perú, Lima (J. Bayona, C. Contreras);; Riga East University Hospital Centre of Tuberculosis and Lung Diseases, Riga, Latvia (V. Leimane);; National Institute of Allergy and Infectious Diseases, National Institutes of Health, Bethesda, Maryland, USA (L.E. Via);; University of Cape Town, Cape Town, South Africa (L.E. Via):; Korean Institute of TB, Seoul, South Korea (H. Kim);; Ministry of Public Health, Bangkok, Thailand (S. Akksilp);; Orel Oblast Tuberculosis Dispensary, Orel, Russia (B.Y. Kazennyy);; Vladimir Oblast Tuberculosis Dispensary, Vladimir, Russia (G.V. Volchenkov);; Taiwan Centers for Disease Control, Taipei, Taiwan (R. Jou); Tartu University Hospital, Tartu, Estonia (K. Kliiman);; Russian Academy of Medical Sciences, Moscow, Russia (O.V. Demikhova)

**Keywords:** multidrug-resistant tuberculosis, second-line drug resistance, tuberculosis and other mycobacterial infections, antimicrobial resistance, bacteria, tuberculosis

## Abstract

Data from a large multicenter observational study of patients with multidrug-resistant tuberculosis (MDR TB) were analyzed to simulate the possible use of 2 new approaches to treatment of MDR TB: a short (9-month) regimen and a bedaquiline-containing regimen. Of 1,254 patients, 952 (75.9%) had no resistance to fluoroquinolones and second-line injectable drugs and thus would qualify as candidates for the 9-month regimen; 302 (24.1%) patients with resistance to a fluoroquinolone or second-line injectable drug would qualify as candidates for a bedaquiline-containing regimen in accordance with published guidelines. Among candidates for the 9-month regimen, standardized drug-susceptibility tests demonstrated susceptibility to a median of 5 (interquartile range 5–6) drugs. Among candidates for bedaquiline, drug-susceptibility tests demonstrated susceptibility to a median of 3 (interquartile range 2–4) drugs; 26% retained susceptibility to <2 drugs. These data may assist national TB programs in planning to implement new drugs and drug regimens.

## Abstract 2

Less toxic and less expensive drugs and shorter treatment regimens are needed.

In 2009, the World Health Organization (WHO) estimated that <5% of patients with multidrug-resistant tuberculosis (MDR TB) were receiving appropriate diagnostic and therapeutic services. This proportion increased to ≈20% by 2012 (*1*,*2*). After decades of relative neglect, health care services for persons with drug-resistant TB are scaling up worldwide at an unprecedented pace (*2*). Part of the delay had been that treatment of MDR TB required a combination of 4–6 expensive, relatively toxic drugs administered for ≈2 years (*3*,*4*). However, treatment guidelines are based on expert opinion, observational studies, in vitro drug-susceptibility testing (DST), and analogies with other mycobacteria because few clinical trials have been conducted of treatment for MDR TB. Treatment success rates average only 55%–65% (*5*–*7*).

Two recent advances have the potential to revolutionize treatment of MDR TB. First, during 2012–2013, two new drugs, bedaquiline and delamanid, were approved provisionally (by the US Food and Drug Administration and the European Medicines Agency, respectively) to treat MDR TB; these drugs are the first truly new anti-TB drugs since rifampin was developed during the 1960s. Because of the urgency of the global MDR TB situation, these drugs were approved on the basis of phase II controlled clinical trials data that used a short-term proxy for long-term treatment outcomes: sputum culture conversion at 2 months (delamanid) or 6 months (bedaquiline). Phase III trials, currently underway, will take years to complete (*8*). The Food and Drug Administration and WHO guidelines recommend adding bedaquiline when an effective regimen (containing at least 4 effective second-line drugs plus pyrazinamide) cannot be designed because of drug toxicity or resistance to fluoroquinolones or second-line injectable drugs (*9*,*10*).

Second, in 2010, Van Deun et al. published their experience with programmatic management of MDR TB in Bangladesh (*11*) and suggested that an intensive 9-month regimen was more effective than treatment success rates reported worldwide and was also considerably less expensive. The regimen consisted of 7 drugs for the first 4 months, followed by 4 drugs for the remaining 5 months of treatment. The results were compelling enough that the regimen is being evaluated in a randomized controlled clinical trial, STREAM (the Evaluation of a Standardised Treatment Regimen of Anti-Tuberculosis Drugs for Patients with MDR TB); results are expected in 2017–2018 (*12*). Because the original study focused on patients without previous use of second-line drugs, both the STREAM trial and countries that are adopting the 9-month regimen exclude patients who have baseline resistance to fluoroquinolones or a second-line injectable drug.

Thus, the exclusion criteria for the 9-month regimen—resistance to a fluoroquinolone or a second-line injectable drug—are virtually the same as the inclusion criteria for the use of bedaquiline. To simulate the extent to which MDR TB patients might qualify either for treatment with a 9-month regimen or for treatment with bedaquiline, we analyzed data from a large observational cohort of patients with MDR TB from 9 countries.

## Methods

### Patient Population

Dalton et al. described the Preserving Effective TB Treatment Study (PETTS) (*13*). In brief, PETTS was a prospective cohort study of consecutive consenting adults who had locally confirmed pulmonary MDR TB and who started treatment with second-line drugs during January 1, 2005–December 31, 2008, in 9 countries (the Philippines, South Africa, Peru, Russia, South Korea, Latvia, Thailand, Taiwan, and Estonia). Patients were treated in accordance with WHO and local treatment guidelines at that time, using regimens of at least 18 months duration, and were followed with monthly sputum cultures throughout treatment (*3*). Eight countries used individualized MDR TB regimens. The study was approved by the US Centers for Disease Control and Prevention (CDC; Atlanta, GA, USA) Institutional Review Board and by institutional review boards at all participating sites. Informed consent for participation in the study was obtained from all patients.

### Laboratory Methods

Baseline isolates of *Mycobacterium tuberculosis* were shipped in batches to CDC for DST using the indirect agar proportion method on Middlebrook 7H10 agar according to the Clinical Laboratory Standards Institute standard as previously described (*13*). DST was conducted for 10 drugs: rifampin (1.0 μg/mL); isoniazid (0.2, 1.0, and 5.0 μg/mL); ethambutol (5.0 μg/mL); ofloxacin (2.0 μg/mL); ciprofloxacin (2.0 μg/mL); kanamycin (5.0 μg/mL); capreomycin (10.0 μg/mL); amikacin (4.0 μg/mL); para-aminosalicylic acid (2.0 μg/mL); and ethionamide (10.0 μg/mL). Cycloserine was not tested at CDC because it requires Lowenstein-Jensen medium, and CDC uses Middlebrook agar. Pyraznamide susceptibility testing and *pncA* sequencing are under way.

CDC’s DST results were used in all analyses. For analysis purposes, ciprofloxacin and ofloxacin were counted as the same drug (“a fluoroquinolone”) and kanamycin and amikacin counted as the same drug (“an aminoglycoside”). High-dose isoniazid resistance was defined as resistance at a concentration of 1.0 μg/mL. 

### Definitions

Second-line injectable drugs were kanamycin, amikacin, and capreomycin. Extensively drug-resistant (XDR) TB was defined as MDR TB plus resistance to any fluoroquinolone and at least 1 of 3 second-line injectable drugs. Pre-XDR TB was defined as MDR TB plus resistance to either any fluoroquinolone or at least 1 of 3 second-line injectable drugs. Effective drugs were defined according to susceptibility demonstrated by CDC’s phenotypic DST results. Standard WHO treatment outcomes were used in all sites (*3*). Treatment success was defined as cure or completion of treatment (*3*). Poor outcome was defined as treatment failure, patient death, or loss to follow-up.

The short regimen was defined as treatment of MDR TB with duration of <12 months (*14*). Candidates for the short regimen were defined as patients whose *M. tuberculosis* isolates had no baseline resistance to fluoroquinolones and second-line injectable drugs. Candidates for bedaquiline-containing regimen were defined as patients whose baseline *M. tuberculosis* isolates had resistance to a fluoroquinolone or second-line injectable drug (i.e., pre-XDR or XDR) (*10*).

### Data Analysis

We conducted statistical analyses using SAS software version 9.3 (SAS Institute Inc., Cary, NC, USA). General descriptive data, including frequencies of basic demographic and clinical variables, were calculated in aggregate and stratified by drug-resistance patterns. Continuous variables were summarized with standard descriptive statistics. We compared proportions using the χ^2^ test or Fisher exact test, as appropriate. Confidence intervals for binomial proportions were calculated by using the 1-sample Wald method. We considered p<0.05 statistically significant.

## Results

A total of 1,659 patients were enrolled in PETTS strictly according to protocol. For 1,254 (75.6%) of these, a baseline *M. tuberculosis* isolate was shipped to, and DST results were available from, CDC. A total of 64.1% of patients were male, and the median age of all patients was 37 (interquartile range [IQR] 28-47) years. HIV co-infection was confirmed in 159 (12.7%) patients, of whom 135 (85%) were in 1 country. A total of 168 (13.4%) patients had new MDR TB; 879 (70.1%) previously received first-line anti-TB drugs; 185 (14.8%) received second-line drugs in the past; and 22 (1.8%) had unknown treatment history. DST was performed in local laboratories in countries to a median of 6 (IQR 4–8) drugs. Overall treatment outcomes were as follows: cure, 659 (52.5%); completion of treatment, 66 (5.2%); treatment failure, 80 (6.4%); death, 170 (13.6%); loss to follow-up, 235 (18.7%); and unknown, 44 (3.5%).

Of the 1,254 patients, 952 (75.9%) had no resistance to fluoroquinolones and second-line injectable drugs and would qualify as candidates for the short regimen on the basis of the drug resistance indication. On the other hand, 302 (24.1%) patients had resistance to a fluoroquinolone or second-line injectable drug and would qualify as candidates for a bedaquiline-containing regimen. The proportion of patients with no baseline resistance to fluoroquinolones and second-line injectable drugs varied among countries from 47.2% to 92.5%; the proportion of patients with pre-XDR or XDR varied from 7.5% to 52.8% (Table 1).

**Table 1:** Proportion of patients with multidrug-resistant tuberculosis who would have qualified for treatment with the short (9-month) regimen or with bedaquiline-containing regimen, 2005–2008*

Candidate regimen	Country, no. patients (%)									
	Estonia, n = 24	Latvia, n = 89	Peru, n = 194	Philippines, n = 386	Russia, n = 96	South Africa, n = 281	South Korea, n = 96	Taiwan, n = 40	Thailand, n = 48	Total, N = 1,254
Short†	13 (54.2)	42 (47.2)	154 (79.4)	357 (92.5)	59 (61.5)	195 (69.4)	58 (60.4)	31 (77.5)	43 (89.6)	952 (75.9)
BDQ‡	11 (45.8)	47 (52.8)	40 (20.6)	29 (7.5)	37 (38.5)	86 (30.6)	38 (39.6)	9 (22.5)	5 (10.4)	302 (24.1)

*Percentages show proportion of patients in cohort form each country who would be candidates for a particular regimen. BDQ, bedaquiline. †Candidates for short regimen were patients without baseline resistance to fluoroquinolones and second-line injectable drugs. ‡Candidates for BDQ-containing regimen were patients with baseline resistance to fluoroquinolones or second-line injectable drugs.

We assessed baseline resistance to individual drugs among potential candidates for the 9-month short regimen and for a bedaquiline-containing regimen and stratified the results by resistance to fluoroquinolones and second-line injectable drugs (Table 2). Susceptibility to high-dose isoniazid was observed in 8.6% of candidates for the short regimen and 3.0% of candidates for a bedaquiline-containing regimen (p<0.001), susceptibility to ethambutol in 41.9% and 27.8% (p<0.001), susceptibility to ethionamide in 81.9% and 80.1% (p = 0.48), and susceptibility to para-aminosalicylic acid in 95.0% and 76.2% (p<0.001), respectively.

**Table 2:** Individual drug resistance in relation to the profile of fluoroquinolone and SLI resistance among patients with multidrug-resistant tuberculosis who could have been candidates for a short (9-month) treatment regimen or a bedaquiline-containing regimen*

Drug, DST result	Total, N = 1,254	Candidate patients, no. (%)				
		Short regimen, FQ-S and SLI-S, n = 952	BDQ-containing regimen			
			FQ-R or SLI-R,† n = 302	FQ-R only, n = 73	SLI-R only, n = 155	XDR, n = 74
INH, high-dose						
NA	11 (0.9)	11 (1.2)	0	0	0	0
R	1,152 (91.9)	859 (90.2)	293 (97.0)	69 (94.5)	154 (99.4)	70 (94.6)
S	91 (7.3)	82 (8.6)	9 (3.0)	4 (5.5)	1 (0.6)	4 (5.4)
EMB						
R	771 (61.5)	553 (58.1)	218 (72.2)	51 (69.9)	113 (72.9)	54 (73)
S	483 (38.5)	399 (41.9)	84 (27.8)	22 (30.1)	42 (27.1)	20 (27)
FQ						
R	147 (11.7)	0	147 (48.7)	73 (100)	0	74 (100)
S	1,107 (88.3)	952 (100)	155 (51.3)	0	155 (100)	0
KAN/AMK						
R	219 (17.5)	0	219 (72.5)	0	150 (96.8)	69 (93.2)
S	1,035 (82.5)	952 (100)	83 (27.5)	73 (100)	5 (3.2)	5 (6.8)
CAP						
NA	9 (0.7)	9 (0.9)	0	0	0	0
R	140 (11.2)	0	140 (46.4)	73 (100)	88 (56.8)	140 (46.4)
S	1,105 (88.1)	943 (99.1)	162 (53.6)	0	67 (43.2)	162 (53.6)
THA						
R	232 (18.5)	172 (18.1)	60 (19.9)	8 (11.0)	30 (19.4)	22 (29.7)
S	1,022 (81.5)	780 (81.9)	242 (80.1)	65 (89.0)	125 (80.6)	52 (70.3)
PAS						
R	120 (9.6)	48 (5.0)	72 (23.8)	13 (17.8)	28 (18.1)	31 (41.9)
S	1,134 (90.4)	904 (95.0)	230 (76.2)	60 (82.2)	127 (81.9)	43 (58.1)

*AMK, amikacin; BDQ, bedaquiline; CAP, capreomycin; DST, drug-susceptibility testing; EMB, ethambutol; FQ, fluoroquinolone; INH, isoniazid; KAN, kanamycin; NA, not available; PAS, para-aminosalicylic acid; R, resistant; S, susceptible; SLI, second-line injectable drug; THA, ethionamide; XDR, extensively drug-resistant. †Comprises FQ-R only, SLI-R only, and XDR cases combined.

Overall, a median of 5 (IQR 5–6) drugs remained effective in candidates for the short regimen and 3 (IQR 2–4) drugs in candidates for a bedaquiline-containing regimen (Table 3). However, in XDR TB cases, a median of only 2 (IQR 1–2) drugs were effective, whereas in pre-XDR cases, a median of 4 (IQR 2–4) drugs were effective (p<0.001). The initial isolate was susceptible to >3 drugs in all of the candidates for the short regimen and in 73.8% (223/302) of candidates for a bedaquiline-containing regimen. Among candidates for a bedaquiline-containing regimen, 90.4% (206/228) of pre-XDR patients had susceptibility to >3 drugs, compared with 23.0% (17/74) of XDR patients (p<0.001).

**Table 3:** Resistance patterns of drugs effective in vitro for multidrug-resistant tuberculosis

	Total, N = 1,254	Candidate effective drugs				
		Short regimen, FQ-S and SLI-S, n = 952	BDQ-containing regimen			
			Combined FQ-R or SLI-R,† n = 302	FQ-R only, n = 73	SLI-R only, n = 155	XDR, n = 74
Median	5	5	3	4	3	2
Interquartile range	4–6	5–6	2–4	4–5	3–4	1–2
Range	0–7	3–7	0–6	2–6	1–5	0–4

*Drug susceptibility test results were analyzed for 7 drugs: high-dose isoniazid, ethambutol, FQ, aminoglycoside, capreomycin, ethionamide, and para-aminosalicylic acid; BDQ, bedaquiline; FQ, fluoroquinolone; R, resistant; S, susceptible; SLI, second-line injectable drug; XDR, extensively drug-resistant. †Includes FQ-R only, SLI-R only, and XDR cases combined.

Several patient characteristics were significantly associated with higher probability of resistance to fluoroquinolones or second-line injectable drugs (and thus candidacy for bedaquiline-containing regimen). These characteristics were unemployment, history of imprisonment, alcohol abuse, history of treatment with second-line drugs, and pulmonary cavities (Table 4).

**Table 4:** Association of patient characteristics with candidacy for a short (9-month) regimen or a BDQ-containing regimen among patients with MDR TB*

Characteristic	Patient candidate, no. (%)			p value
	Total	Short regimen	BDQ-containing regimen	
Sex				
M	804	613 (76.2)	191 (23.8)	0.72
F	450	339 (75.3)	111 (24.7)	
Employment status				
Unemployed	475	328 (69.1)	147 (30.9)	**<0.001**
Disabled, retired, student, housewife	192	150 (78.1)	42 (21.9)	0.37
Employed	581	471 (81.1)	110 (18.9)	
History of imprisonment				
Yes	81	50 (61.7)	31 (38.3)	**0.001**
Unknown	181	130 (71.8)	51 (28.2)	0.08
No	992	772 (77.8)	220 (22.2)	
Homeless				
Yes	29	21 (72.4)	8 (27.6)	0.54
Unknown	137	91 (66.4)	46 (33.6)	**0.005**
No	1,088	840 (77.2)	248 (22.8)	
Alcohol abuse				
Yes	186	108 (58.1)	78 (41.9)	**<0.001**
Unknown	52	31 (59.6)	21 (40.4)	**<0.001**
No	1,016	813 (80)	203 (20)	
HIV status				
Positive	159	108 (67.9)	51 (32.1)	0.88
Unknown	468	422 (90.2)	46 (9.8)	**<0.001**
Negative	627	422 (67.3)	205 (32.7)	
Any co-morbidity other than HIV infection				
Yes	335	257 (76.7)	78 (23.3)	0.69
No	919	695 (75.6)	224 (24.4)	
Classification by prior treatment history				
Received first-line drugs	879	719 (81.8)	160 (18.2)	**<0.001**
Received second-line injectable drugs	185	103 (55.7)	82 (44.3)	**0.04**
New case	168	112 (66.7)	56 (33.3)	
Classification by prior TB treatment outcome				
Relapse	181	124 (68.5)	57 (31.5)	0.66
Failure, loss to follow up, change to second-line regimen	598	444 (74.2)	154 (25.8)	**0.04**
Chronic	190	172 (90.5)	18 (9.5)	**<0.001**
Unknown	119	102 (85.7)	17 (14.3)	**<0.001**
New case	166	110 (66.3)	56 (33.7)	
Body mass index				
<18.5	471	369 (78.3)	102 (21.7)	0.12
>18.5	783	583 (74.5)	200 (25.5)	
Site of TB disease				
Extrapulmonary and pulmonary	57	45 (78.9)	12 (21.1)	0.59
Pulmonary only	1,195	906 (75.8)	289 (24.2)	
Bilateral TB disease				
Yes	993	748 (75.3)	245 (24.7)	0.48
No	236	183 (77.5)	53 (22.5)	
Cavities on chest radiograph				
Unilateral	477	339 (71.1)	138 (28.9)	**<0.001**
Bilateral	293	210 (71.7)	83 (28.3)	**<0.001**
No	484	403 (83.3)	81 (16.7)	
AFB smear at the start of MDR treatment				
Positive	1,063	810 (76.2)	253 (23.8)	0.37
Negative	155	113 (72.9)	42 (27.1)	

*Row percentages show proportion of patients with each characteristic that would be candidates for a particular regimen. AFB, acid-fast bacilli; BDQ, bedaquiline; MDR, multidrug-resistant; TB, tuberculosis.Bold type indicates statistical significance.

Overall treatment success among candidates for a short regimen was 66.1% (95% CI 63.0%–69.1%), varying from 90% to 50% among countries. Among candidates for a bedaquiline-containing regimen, treatment success was 39.9% (95% CI 34.3%–45.6%), varying from 90% to 10% among countries. 

## Discussion

Data from our large prospective observational cohort study of 1,254 MDR TB patients showed that about three fourths would qualify as candidates for the short regimen and about one fourth would qualify as candidates for a bedaquiline-containing regimen on the basis of the drug resistance indication. DST demonstrated susceptibility to a median of 5 (IQR 5–6) drugs among candidates for the 9-month regimen, whereas among candidates for bedaquiline, a median of 3 (IQR 2–4) drugs remained effective. Overall treatment success was 66% for candidates for the short regimen and 40% for candidates for a bedaquiline-containing regimen. This analysis has several practical implications for national TB control programs that plan to start using short regimens or bedaquiline for programmatic management of drug-resistant tuberculosis.

First, data from this report may assist national TB control programs in planning the number of patients to be enrolled for short or bedaquiline-containing regimen. Not all countries have representative data from routine drug resistance surveillance or surveys; thus, data from this report can be used to estimate numbers of candidate patients for certain regimens. The 2013 WHO report included combined representative data from 75 countries and 4 territories showing that the 32% of persons had MDR TB resistant to a fluoroquinolone, a second-line injectable drug, or both, and thus these persons might be eligible to receive bedaquiline (*1*). Our data showed a relatively similar proportion (26%). The remaining 74% of patients could be considered as candidates for the short regimen. However, the proportions of patients in our study who might qualify for the 9-month or bedaquiline-containing regimen varied widely among countries: 47%–93% and 8%–53%, respectively. This variation in baseline drug resistance probably reflected previous use of anti-TB second-line drugs in these countries and the consequent epidemiology of MDR TB (*13*).

Second, we report on the prevalence of resistance to first- and second-line drugs by the status of resistance to fluoroquinolones or second-line injectable drugs. Candidates for the short regimen had, on average, susceptibility to 5 anti-TB drugs. We did not have DST for pyrazinamide and clofazimine (which are part of the 9-month regimen). Susceptibility to high-dose isoniazid, also included in the short regimen, was found in only 8.6% of candidates for that regimen. To our knowledge, only 1 randomized clinical trial has documented improved interim treatment outcomes (reduced time to sputum culture conversion and higher proportion with sputum culture negative 6 months after treatment started) among patients receiving high-dose isoniazid (16–18 mg/kg) as an adjuvant to second-line drugs in documented MDR TB (*15*). More clinical research is needed about use of high-dose isoniazid to treat MDR TB (*16*). Susceptibility to ethambutol was found in 42% and to thioamides in 82% of candidates for the short regimen. On the other hand, candidates for a bedaquiline-containing regimen frequently had resistance to other first- and second-line drugs with a median of 3 drugs that remained effective; the number of effective drugs progressively decreased from 3 or 4 in patients with pre-XDR to 2 drugs in XDR TB. Therefore, countries planning to implement bedaquiline for programmatic use should include plans to procure third-line drugs, such as linezolid and clofazimine. These additional drugs are needed to build regimens with the minimum 4 effective drugs because of the extent of resistance to other second-line drugs among patients who might qualify for bedaquiline. Alternatively, this study’s results can be used to infer what might happen if bedaquiline were introduced solely on the basis of the result of fluoroquinolone or second-line injectable drug resistance. Without the capacity for full-spectrum DST, widespread acquired bedaquiline resistance would be expected quickly.

Finally, when countries implement the short regimen or bedaquiline under programmatic conditions, a comparator arm is unlikely to be evaluated to determine the effectiveness of the regimens. Patients placed on a standard WHO MDR TB regimen will not be an appropriate control group to make valid comparisons. In this study, overall treatment success was 66% among candidates for the short regimen and 40% among candidates for a bedaquiline-containing regimen. A meta-analysis of the 18- to 24-month regimen outcomes reported by Falzon et al. (*17*) included data from 26 treatment centers and reported treatment success of 64% among 4,763 patients with MDR TB and no additional resistance to fluoroquinolones or second-line injectable drugs (this group of patients would be most comparable to candidates for the short regimen) and 40%–56% among patients with additional resistance to second-line injectable drugs or fluoroquinolones and with XDR TB (similar to candidates for bedaquiline). Thus, the proportions of patients with treatment success in the respective groups defined by drug resistance status can be used as historical comparators to assess the effectiveness of treatment in cohorts on the short regimen or the bedaquiline-containing regimen. Also, these studies clearly demonstrate the tremendous capacity for improvement of MDR TB treatment outcomes; new drugs and regimens are desperately needed.

Our report emphasizes the crucial need for strong laboratory capacity to manage drug-resistant TB. A few years ago, the Xpert MTB/RIF assay (Cepheid Inc., Sunnyvale, CA, USA) for detecting *M. tuberculosis* and rifampin resistance became widely available. Many countries are adopting this diagnostic tool and thus increasingly diagnosing MDR TB. However, for implementation and programmatic use of short and bedaquiline-containing regimens, national TB control programs would need capacity for rapid, accurate DST for both first- and second-line drugs to appropriately select candidates for the respective regimens and ensure the availability of at least 3 other effective drugs. This need should be considered during planning of the implementation of these new regimens.

Our study findings are subject to several major limitations in interpretation and applicability to programs. This study did not evaluate the frequency of drug-related toxicities leading to permanent discontinuation of fluoroquinolones or second-line injectable drugs. These patients would be candidates for bedaquiline-containing regimens even if their isolates remained susceptible to these drugs. Thus, we might have underestimated the proportion of patients eligible for bedaquiline. DST results were not available for pyrazinamide by the time of this analysis. On the basis of published data, pyrazinamide resistance in patients with MDR TB in countries included in PETTS varied from 49% in Thailand, 52% in South Africa, and 55% in Taiwan to 85% in South Korea (*18*–*21*). Thus, our study might overestimate the proportion of patients with MDR TB who would respond to the 9-month regimen because >50% of isolates could be resistant to pyrazinamide. CDC did not test for moxifloxacin when this work was conducted because standardized procedures had not yet been established. CDC does not test for clofazimine because standardized procedures using currently available technology have not been established. CDC does not test for cycloserine, which requires Lowenstein-Jensen medium. The intrinsic reproducibility of phenotypic DST for several drugs tested routinely including ethambutol, thioamides, cycloserine, and para-aminosalicylic acid is poor. All patients in the PETTS study were treated under WHO-recommended regimens during 2005–2010, when new treatment options were not available; thus, treatment outcomes might not be able to be extrapolated to new regimens.

Current standards for treating patients with MDR TB pose at least 2 major problems that reduce the effectiveness of treatment and treatment success rates. First, the long duration of treatment—a total of at least 20 months—places a major burden on patients and health care systems. Second, common serious adverse drug reactions contribute to reduced adherence or suspension of treatment. Given the global prevalence of MDR TB and low treatment success rates, better TB drugs and shorter regimens are urgently needed to enable more effective, less toxic, and less expensive treatment for persons with MDR TB.
